# Exons 19 and 21 of Epidermal Growth Factor Receptor Are Highly Conserved in Squamous Cell Cancer of the Head and Neck

**DOI:** 10.1155/2009/649615

**Published:** 2010-01-05

**Authors:** Matthew Carlson, Beverly Wuertz, Jizhen Lin, Randy Taylor, Frank Ondrey

**Affiliations:** Department of Otolaryngology, University of Minnesota, MMC 396, 420 Delaware ST SE, Minneapolis, MN 55455, USA

## Abstract

*Objective*. Epidermal growth factor receptor (EGFR) tyrosine kinase inhibition (TKI) is a promising treatment in upper aerodigestive malignancies. EGFR inhibitors might be more effective in patients whose tumors harbor specific EGFR mutations. The presence of specific EFGR mutations is predictive of over a 75% response rate to TKI therapies as compared to 10% in wild type cases of non-small cell lung cancer. Our objective was to examine whether these mutations might occur in upper aerodigestive cancers. *Design*. DNA was extracted from 20 head and neck squamous cell tumors and 4 squamous cell carcinoma cell lines and sequenced the receptor using published primer pairs. We then compared the results against published mutations. *Results*. No exon 19 or 21 mutations were found in any of the 20 tumors and 0 of 4 cell lines. Based on the tumor data we would predict that no greater than 8% of head and neck tumors (CI 97.5%) would be likely to harbor either of these mutations. *Conclusions*. Our findings are comparable to results recently published of Korean, Austrian, and Spanish patient populations and we conclude that exon 19 and 21 EGFR mutations are not more common in head and neck cancer than in nonsmall-cell carcinoma.

## 1. Introduction

Upper aerodigestive cancers including oral cavity, pharynx, and laryngeal malignancies affects an estimated 3 million patients worldwide yearly [1]. Carcinogenesis has been linked to the overuse of tobacco and alcohol [2]; more recently the human papillomavirus has been implicated [3]. Unfortunately, the cure rates for these malignancies have remained stagnant for several decades, and so new therapies for this malignancy are integral to controlling this disease. 

Over the past decade, the emergence of targeted therapies represents a new development in the treatment of solid tumor malignancies [4–10]. Several agent classes have been used to improve survival in a variety of tumor types, and EGFR-disruptive therapies have had promising results in lung cancer in specific patient populations [11–14]. In head and neck carcinoma, EGFR directed antibody therapy has been approved for simultaneous use with radiation therapy for improved survival of advanced stage disease [15]. Other clinical trials with these agents suggest response rates for recurrent or metastatic head and neck cancer may be similar to studies in lung cancer [16, 17]. 

In lung cancer, it has recently been reported that tumors harboring mutations of exon 19 and 21 were associated with improved clinical response rates [18]. In the present study, we examined 20 head and neck cancer specimens and 4 cell lines for mutations in either exon and report here the high conservation of these sequences of the EGFR gene.

## 2. Materials and Methods

Tumor specimens were obtained through the University of Minnesota tissue procurement facility in compliance under University of Minnesota Institutional Review Board approval. To remain within human investigation HIPAA compliant guidelines established for the tissue procurement facility, these tumors were supplied without patient identifying data. The tumors were derived from primary cancers of the upper aerodigestive tact (oral cavity, oropharynx, larynx, and hypopharynx). Head and neck cancer demographics for our university patient population are typical: 2 : 1 male to female preponderance, average age = 60 years. Approximately 5% of the tumors in our tumor bank are from underrepresented minority populations, a reflection of our regional referral base. Between 2002 and 2003, 20 randomly selected frozen specimens from patients with pathologically confirmed head and neck squamous cell carcinoma (HNSCC), along with 4 squamous carcinoma cell lines (CA-9-22, NA, UM-SCC-9, UM-SCC-11B), were obtained for DNA analysis. 

For mutational analysis of the EGFR coding sequence for exons 19 and 21, DNA extraction was performed using the Wizard Genomic DNA Purification Kit (Promega, Madison, WI) according to the manufacturer's instructions. Forward and reverse primers were made for both of the EGFR mutational hotspots located on exons 19 and 21. Previously published primer sequences [18] were synthesized by Integrated DNA Technologies (Coralville, IA). PCR amplification was carried out using AmpliTaq Gold Polymerase (Applied Biosystems, Foster City, CA) and an MJ Research thermocycler (Bio-Rad, Hercules, CA). PCR cycling parameters were 95°C hot start for 9 minutes, then 94°C for 30 seconds, 60°C for 1 minutes, and 72°C for 45 seconds for a total of 25 cycles, followed by a final extension step of 72°C for 5 minutes. PCR product purification was carried out using Purelink PCR Purification Kit (Invitrogen, Carlsbad, CA). UV spectrophotometry was then used to assure quality and calculate DNA sample concentration. The resulting DNA was then sent for sequencing at the University of Minnesota Microchemical Sequencing facility. Sequencing results were viewed and compared with published sequences using Chromas software (Technelysium Pty Ltd, Tewantin QLD, Australia). 

### 2.1. Statistics

The Fisher Exact test was used to compare the published frequency of mutations in a recently published lung cancer study using Prism GraphPad 4 Software (GraphPad, San Diego, CA). To calculate the 95% confidence interval (CI) for a proportion, we used the exact method for a binomial parameter [19].

## 3. Results

High-quality DNA was isolated from all 20 tumor samples and 4 cell lines. PCR analysis of exons 19 and 21 yielded 100% homology between the nucleotide sequences in question. No mutations were found in the 20 tumor samples or the four cell lines. There was no statistical difference (*P* = .5) between published rates of EGFR mutations in NSCLC [18] and our data. Next, we calculated a 95% confidence interval based on the presence of 0% mutations in either exon 19 or 21. Based on a 0% mutation rate, we would predict that no greater than 17% of head and neck tumors would be likely to harbor mutations at exon 19 or 21.

## 4. Discussion

These data demonstrate that only a small number of head and neck tumors are likely to have mutations of EGFR receptor exons 19 and 21. Mutations of exons 19 and 21 are the most common EGFR receptor mutations in lung cancer and confer receptor tyrosine kinase activation [11, 18]. It is postulated that tumors harboring EGFR mutations demonstrate inflexible “tumor addiction” to the high EGFR transduction environment since they were born out of this condition. TKIs disrupt the primary prosurvival signaling pathway and lead to rapid tumor cell death [20]. A number of recent studies in head and neck cancer demonstrate a low, but significant, level of clinical response (up to 12%) of single agent EGFR TKI, in a setting of recurrent or metastatic disease [21, 22]. Since activating EGFR mutations at exons 19 and 21 is highly associated with a good clinical response to EGFR inhibitors in lung cancer, the fact that our tumor specimens do not demonstrate these mutations may support the relatively low levels of clinical response in recent head and neck cancer clinical studies. Since 0% of our specimens and cell lines were associated with mutations, we extrapolated a 95% confidence interval for the likely number of head and neck tumor specimens in a similar large population of U.S. head and neck cancer patients. We would predict that no more than 17% of head and neck tumors would be likely to harbor a mutation at either exon 19 or 21. These results are comparable to 3 small studies demonstrating that EGFR mutations occur in very few-sampled HNSCC in Korean [23], Austrian [24], and Spanish [25] patients. Further, our data also predicts that these mutations should not occur more often than similar mutations in NSCLC. 

Since TKI therapy is expensive and primarily benefits a minority of patients receiving it as a single agent, it would be very useful to predict which patients are most likely to receive benefit. Because these agents are most successful in patients whose tumors harbor activating mutations, empiric TKI therapy for all patients with recurrent or metastatic head and neck cancer is impractical. Screening patients for TKI sensitive mutations may allow for more efficient therapy and improved patient outcomes. 

The tumor patients in our population are fairly typical head and neck cancer patients when segregated for age, gender, and tobacco use. However, the location of our cancer center in the U.S. Midwest has relatively low numbers of non-Caucasian patients in our clinical trials and specimen banks pool (approximately 5%), a number that reflects the demographics of our 4 state referral pattern. Another limitation to our study is the fact that the tumor specimens are supplied without identifying data from the patients, per human investigation regulations of the tumor bank. In spite of these limitations, it is helpful to know that since no mutations exist at either locus in our samples, it might suggest that only a minority of such patients will likely respond to current EGFR-directed therapies. Further investigations in HNSCC correlating exons 19 and 21 mutation status and TKI response are required.

## Figures and Tables

**Figure 1:**
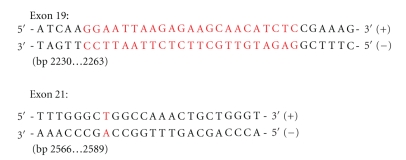
Mutational hotspots in EGFR gene clustering at ATP-binding pocket. The above sequences contain the 2 primary sites of interest for mutation analysis. The areas in red represent the regions where mutations most often occur. The mutation in exon 19 is most commonly a deletion mutation, while the mutation seen in exon 21 is a missense mutation (T → G substitution at nucleotide 2573 resulting in a L858R amino acid substitution).

**Figure 2:**
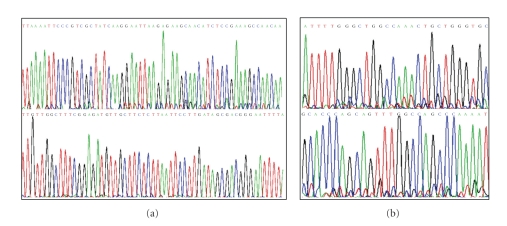
Chromatograms of PCR products. (a) graphs displaying exon 19 sense and antisense strands on top and bottom, respectively. (b) Graphs displaying exon 21 sense and antisense strands on top and bottom respectively. In both exons 19 and 21 no significant double peaks were obtained signifying the absence of any mutations within these reading frames.
